# Visualising adoption processes through a stepwise framework: A case study of mechanisation on the Nepal Terai

**DOI:** 10.1016/j.agsy.2021.103200

**Published:** 2021-08

**Authors:** Brendan Brown, Gokul P. Paudel, Timothy J. Krupnik

**Affiliations:** a International Maize and Wheat Improvement Center (CIMMYT), Kathmandu, Nepal; b International Maize and Wheat Improvement Center (CIMMYT), Dhaka, Bangladesh

**Keywords:** Appropriate agricultural mechanisation, Mechanised harvesting, Zero tillage, Sustainable intensification, Adoption processes

## Abstract

**CONTEXT:**

The desire for agricultural mechanisation is mainstreaming across the Global South, yet there are limited tools through which to monitor and estimate progress made in pursuit of this. Despite Nepal enacting an agricultural development agenda focused on mechanisation to address issues of productivity, labour scarcity, inclusive economic growth and sustainability, it remains one of the few places in South Asia that is yet to see substantial agricultural mechanisation rates. We use this scenario as a case study to propose and investigate adoption processes.

**OBJECTIVE:**

This research aims to provide a baseline to understand progress made towards Agri-mechanisation on the Nepal Terai. Despite decades of promotional efforts, there are only limited comprehensive analyses of the status of agricultural mechanisation in Nepal that cover diverse machinery and go beyond binary adoption estimates, nor a framework to understand different types of (non-)adopters.

**METHODS:**

The applied non-binary ‘Stepwise Process of Mechanisation’ framework provides a systematic process for investigation of the status of agricultural mechanisation on the Nepal Terai. This framework is applied to representative survey data from 14 districts across 1569 households from Nepal's Plains (Terai) region.

**RESULTS AND CONCLUSIONS:**

Results suggest that decades of activity have not yet led to the substantial closure of exposure gaps, nor sufficient ownership of machines that enables accessible fee-for-hire service provision. Exposure gaps were substantial in all machines, meaning current demonstration programs may not be achieving their targeted outcomes. Across nearly all machinery, a primary reason for limited progression to sustained adoption was a lack of service providers, a manifestation of limited machinery ownership, meaning current broad subsidy programs aimed at procurement may not be achieving intended outcomes. However, substantial pools of potential adopters and concentration of supply-side constraints highlight that with targeted intervention, rapid rural mechanisation is possible in the near future on the Nepal Terai.

**SIGNIFICANCE:**

This research provides a foundation on which to understand the progress made towards small holder agricultural mechanisation. For the first time in South Asia, a systematic analysis through a novel stepwise framework has clarified and updated the status of agricultural mechanisation on the Nepal Terai. This work also lays the foundation for future work to explore the drivers, implications and inclusiveness of agri-mechanisation, utilising the identified typologies, both in Nepal and more broadly where increased nuance in understanding the status of agricultural mechanisation is warranted.

## Highlights

• Few ways to measure agri-mechanisation progress exist leading to limited assessment of decades of activity in Nepal
• We apply a novel adoption framework to create a baseline of agri- mechanisation progress across the Nepal Terai
• Substantial exposure gaps and limited machinery ownership create a large pool of constrained potential adopters
• While limited to date, Nepal has the potential to rapidly mechanise if exposure gaps and usage constraints are ameliorated
• A method through which to understand adoption processes, drivers, status and implications is provided for future studies

## Graphical abstract

**Figure:**
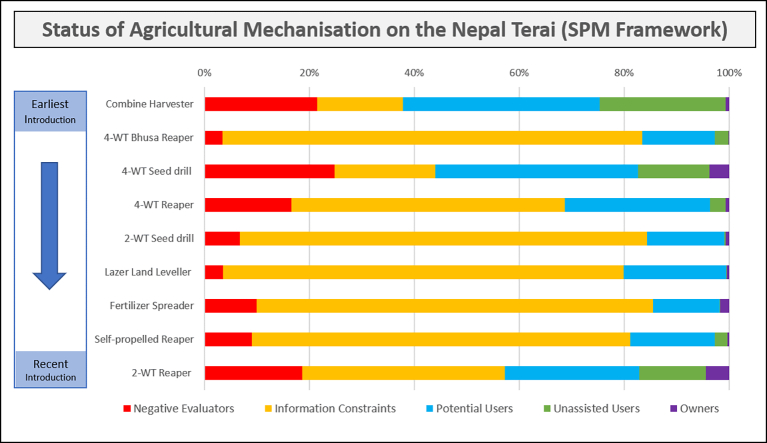

## 1 Introduction

Agriculture can be a key source of economic growth and poverty reduction, as well as a pathway to environmental sustainability in South Asia (Gauchan and Shrestha, 2017; Gathala et al., 2021). Yet in Nepal, two thirds of farmers are subsistence-based and more than half of Nepal's districts face regular food insecurity (Paudel et al., 2019). Compounding this, there is growing agricultural labour scarcity due in large part to rural out-migration, especially in the Plains (Terai) region that borders northern India where opportunities for non -farm income are increasingly lucrative in comparison to agricultural activities (Maharjan et al., 2020). This creates a clear and urgent need to address agricultural productivity, profitability, and sustainability to ensure the viability of rural livelihoods in Nepal. In response, a consensus has emerged on the merits of increased commercial integration of smallholder farming systems driven through agricultural mechanisation (Gauchan and Shrestha, 2017; Paudel et al., 2019), but with a focus on sustainable intensifications that fit within smallholder contexts and ensure limited externalities associated with agricultural intensification (Gathala et al., 2021).

In 2014, Nepal's first comprehensive policy focusing on agricultural mechanisation as a part of a twenty-year agricultural development strategy was released (Devkota et al., 2020) and over the last decade, there has been a growing investment in agricultural mechanisation by both the Government of Nepal (e.g. the Prime Minister's Agricultural Modernisation Program - PMAMP) and among donors (e.g. the Cereal Systems Initiative for South Asia). Yet despite substantial efforts, a clear picture of the status of mechanisation across Nepal's Terai – the low elevation and comparatively high productivity portion of the country– remains absent. This is particularly notable given the otherwise substantial mechanisation of agricultural production systems across other parts of South Asia. Such questions are also relevant to the emerging narrative across the Global South, and particularly sub-Saharan Africa, on the need for mechanisation of agricultural production systems yet the slower than expected rate of mechanisation, and the limited avenues to explore and understand such mechanisation processes (Kahan et al., 2018; Ayele, 2021).

Two of the few quantifications of mechanisation status in Nepal include the study by Biggs et al. (2011), which indicated that the total number of tractors in 2010 was 42,000, of which 71% where four wheeled, which is roughly consistent with the study by Diao et al. (2020) who estimated in 2016 there were 47,000 tractors in Nepal of which 64% were four wheeled. Yet tractor ownership alone does not facilitate a true understanding of agricultural mechanisation more broadly, given the various tractor utilisation purposes and often limited degree to which they are involved in agriculture (Pradhan et al., 2016). Compounding this knowledge gap, there is only limited exploration of the uptake of a diverse range of agricultural machinery due to limitations in national census data (Diao et al., 2020). The trend is true also in other areas of emerging interest such as sub-Saharan Africa where tractors per unit area are used as a metric for quantifying agricultural mechanisation (Daum and Birner, 2020). Hence, a clear understanding of the broader status of agricultural mechanisation in Nepal's Terai and more widely across various global regions remains absent.

Understanding progress towards agricultural mechanisation is potentially useful in enabling a reflection on the effectiveness of various methods used for promotion, to plan for future interventions and to enable further investigations on the inclusiveness of agricultural mechanisation achieved. This is particularly important as there are many ways to promote agricultural technologies in any given context. In Nepal, a clear example of this is the ten year objective of the PMAMP to provide a 50% capital subsidy for the purchase of selected agricultural equipment and tools (Devkota et al., 2020). However Paudel et al. (2020), suggest that this policy may not be adequate to enable increased adoption and the sustained use of appropriate farming machinery in Nepal without other support mechanisms. This includes a need for renewed focus on extension efforts to overcome exposure gaps with potential adopters and access to infrastructure, without which subsidy programs will primarily benefit larger, wealthier farmers and disadvantage resource poor smallholder farmers in Nepal (Freshley and Delgado-Serrano, 2020).

Yet few multi-machinery investigations exist that also explore beyond binary rates of adoption. More recently, some methods have been proposed, such as the Process of Agricultural Utilisation Framework (PAUF), that as applied to Conservation Agriculture in Africa, highlighted crucial issues in adoption of a set of intensification technologies (Brown et al., 2017b). This research adapts the PAUF to shift focus from a set of agronomic practices to machinery usage, with an additional focus on ownership and supply side vs. demand side negative evaluations. Such adaptation increases the utility of the framework to be applied to a wider range of agricultural change processes beyond agronomic changes, and may be further applied to contexts outside of Nepal where suitable data is available.

Such adaptions are also important in the context of the recent drive to prioritise mechanisation and scaling studies in the literature to overcome the empirical vacuum created after failed state-led mechanisation programs (Daum and Birner, 2020). This adapted framework can provide a basis for exploration of various elements of other recently proposed frameworks, such to address multiple elements of the Ecological Intensification framework (Kernecker et al., 2021), to assist in stage five (monitoring and evaluation) of the scaling Readiness approach (Sartas et al., 2020) or even to explore areas of inertia and farmer decision making in the PROMIS framework (Wigboldus et al., 2016). The framework also supports the exploration of Adoption pathway analysis (Montes de Oca Munguia et al., 2021) to quantify beyond binary adoption and could be used as an additional tool to support ADOPT (Kuehne et al., 2017) implementation though deeper segmentation of different types of adoption and non-adoption. It has the potential to provide additional utility to these frameworks through it's novel segmentation of mechanisation and adoption processes.

The purpose of this paper is to propose and apply the Stepwise Process of Mechanisation (SPM) framework as an adapted PAUF to understand and visualise the status of adoption of various agricultural machinery in Nepal. This is proposed as an initial step towards understanding the progress, drivers and implications of smallholder agricultural mechanisation on the Nepal Terai. This is the first time a non-binary framework such as the PAUF is applied in South Asia, which aims to deepen understanding of adoption processes in, and the mechanisation status of, Nepal.

**Fig. 1:**
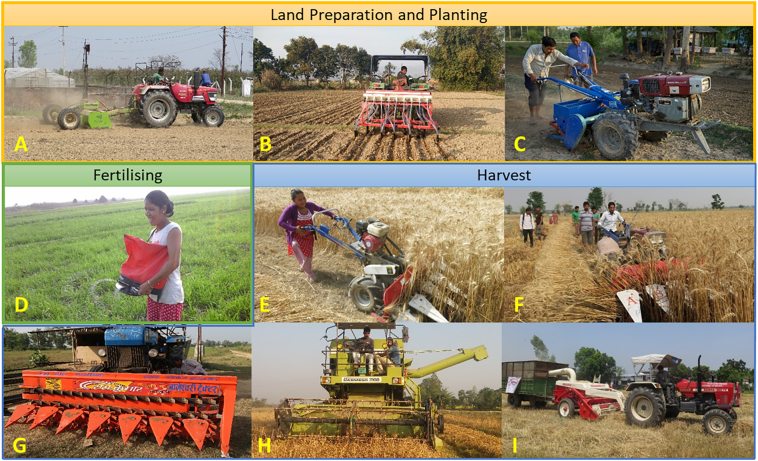
Nine ‘sustainable intensification’ agricultural mechanisation technologies investigated in this study: [A] Laser Land Leveller; [B] four-wheel tractor (4WT) seed drill; [C] a two-wheeled (2WT) seed drill; [D Fertilizer Spreader; [E]Self Propelled Reaper; [F] 2WT attachable reaper; [G] 4WT attachable reaper; [H] Combine Harvester; and [I] Bhusa Straw Reaper. Photo Credit: CSISA Project team/CIMMYT.

This study presents a theoretical framework to understand the current status of adoption of a diverse set of agricultural machinery in Nepal. Applying an adapted PAUF (Brown et al., 2017b), the status of machinery adoption among 1570 farmers is examined, with representative coverage across 14 out of 20 of Nepal's Terai districts. Focus is placed on nine commonly promoted farm machines (Fig. 1) that are consistent with the ‘sustainable intensification’ agenda as emphasized by many development and governmental programs. In doing so, a visualisation of progress is possible that sets a baseline through which to further reflect, understand and plan for future interventions in the agricultural mechanisation sector. This is particularly timely given the challenges and policy opportunities created through the administrative restructuring resulting from Nepal's new 2015 constitution and the empowerment of provincial and local governments to implement agricultural extension programs. Hence, we provide not only a theoretical framework for monitoring progress towards mechanisation objectives, but a building block for further analysis of Nepal's mechanisation progress, drivers and implications. Such an approach is also applicable more widely to understand and plan for agricultural mechanisation.

## 2 Materials and methods

### 2.1 Technologies investigated

Nine mechanisation technologies representing prominently promoted machineries across the Nepal Terai over the past three decades (through the Rice-Wheat Consortium, Cereal System Intensification for South Asia and Government programs, among others) were selected for analysis. This consists of three planting related machines, one seed and fertilizer application machine and five harvesting or post-harvest machines (Fig. 1). These machines were specifically selected for their categorization under the umbrella of ‘sustainable intensification’, noting they are unlikely to lead to unsustainable agronomic and environmental outcomes (hence the exclusion of rotovator, cultivator and disk harrow), are relevant in the Nepal Terai (hence the exclusion of the mini-tiller which are more prominent in the hill districts) and with a focus on agronomic machines (hence the exclusion of irrigation pumps). Descriptions and the historical context for each technology are outlined in Table 1.

**Table 1:** Description and historical context of the nine investigated machines. Photos of each machine are given in Fig. 1. Introduction year is estimated based on Project and government documentation. Project documentation from CSISA (2020) provides the majority of estimate due to otherwise unavailable estimation.

Category	Machine	Introduction year (Nepal)	Description	Historical context/ Nepal
Preparation and Planting	Laser Land Leveller	2011	A Laser Land Leveller (LLL) is typically fitted behind a four-wheel tractor (4WT) and utilized as part of periodic field preparation. Using a laser-guided leveling system, soil is corrected for micro-elevation differences within plots (Jat et al., 2009). This enables uniform water and nutrient distribution and increases water, energy, and nutrient use efficiency, and ultimately crop productivity, while reducing costs (Aryal et al., 2015)	The LLL was introduced through project-based demonstrations, although this technology has been prevalent in North-West India since 2002. Despite a governmental subsidy and promotional activities, until 2019 only 134 units of LLL are in operation in Nepal and most them are subsidized by either public or developmental sectors (CSISA, 2020).
	4WT Seed drill	1994	A 4WT seed drill is attached behind a four-wheel tractor and used for precision planting of primarily cereal and pulse crops. Many of these drills can be used for zero- or reduced-tillage (Gupta and Seth, 2007). Multiple line sowing with proper seed and fertilizer application is a key feature of seed drill, which has been shown to have multiple benefits in Nepal (Gathala et al., 2020).	4WT seed drills have been tested in Nepal with a primary focus on on-station technology validation. Promotion of 4WT seed drills took place only after 2009, through various projects. Until 2019, about 200 4WT seed drills are in use on the Nepal Terai (CSISA, 2020).
	2WT Seed drill	2010	A two-wheel tractor (2WT) seed drills is attached behind a two-wheel tractor or power tiller and operate similar to 4WT seed drills. However, 2WT seed drills are considerably less expensive, and more versatile on small plots and in smallholders farming systems, though they have more limited field capacity. This machine can also be used for strip tillage.	2WT seed drills are relatively new in Nepal, although 2WT are common with around 20,000 two-wheel tractors operational in Nepal (CSISA, 2020). There have been multiple versions of the 2WT seed drill in Nepal developed through various research initiatives, with the complete seed and fertilizer 2WT seed drill introduced in 2010. Currently there approximately 100 seed drills of this type operational on the Nepal Terai (CSISA, 2020).
Fertilizer	Fertilizer Spreader	2012	A spreader contains a bag that can carry approximately 10 kg of fertilizer or seed. The bottom of the bags contains circular disc connected with hand crank that distributes fertilizer or seed equally when manually rotated. This reduces patchy distribution, plot variability and low yield common with hand broadcasting, and can assist in reducing application time (Park et al., 2018).	While primarily project driven, some importers have demonstrated this technology directly with farmers. There are almost 1000 spreaders adopted by the farmers in the rice-wheat cropping systems in Nepal Terai (pers comm). Most of these spreaders adopted by the farmers are subsidized by the Government of Nepal. Spreaders in Nepal were originally imported from USA, but are increasingly from China in recent years (Biggs et al., 2011). Unlike the other investigated machinery, subsidies are limited for the spreader.
Harvest	Self-propelled Reaper	2014	The self-propelled reaper is a standalone machine used for harvesting rice or wheat and cannot be attached to any existing tractor or power tiller. Compared with the manual harvesting, it reduces time and cost of harvesting which often falls to females, leading to its proclamation as female friendly (Paudel et al., 2018). The self-propelled reaper can harvest crops in very small plots and with some adjustments in plots with waterlogged conditions.	Self-propelled reapers were project introduced in Nepal's western Terai, although this technology has been present in east Asian countries for decades. By 2020 over 300 reapers are in operation, mostly in Nepal's western Terai, with farmers who use crop residue for livestock most likely to preference this machine (CSISA, 2020).
	2WT Reaper	2014	The 2WT reaper functions similarly to the self-propelled reaper, though it functions through attachment to a 2WT. This provides increased utility as it can be removed after completing harvest. These types of reaper are preferred by small to medium type of farmers due to small land holding (Paudel et al., 2018).	Although different models of reapers were promoted since the 1990s, few were popular among farmers until recently, and in 2019 over 3500 reapers are in operation in Nepal (CSISA, 2020). Different public organizations including the Department of Agriculture and the private-sector led Nepal Agricultural Machinery Entrepreneurship Association have been active in increasing the availability of the 2WT reaper (CSISA, 2020).
	4WT Reaper	2002	4WT reapers are functionally similar to smaller reapers, though they attach to 4WT tractors.	The 4WT reaper is one of the few investigated technologies that has been led by independent farmer adoption. This technology was common in bordering districts of India and eventually permeated the border regions of the Nepal Terai. There are over 200 4WT reapers in use in Nepal (CSISA, 2020).The popularity of this technology is diminishing, however, due to the use of the combine harvesters and yield losses due to grain shattering during harvest.
	Combine Harvester	1990	Unlike reapers which only cut the crop, combine harvesters both cut and thresh crops at the same time. This negates the need for independent threshing, reduces harvesting costs and time (Paudel et al., 2015). Farmers who own the large farms prefer this technology, while livestock owners generally do not (Paudel et al., 2015).	The first combine harvester was introduced in Nepal Terai via permeation from the Indian border through local information and service networks, in the main without agricultural development project intervention. Almost 650 combine harvesters were used in the Nepal Terai during 2019, with over half coming from India during Nepal's peak rice and wheat harvesting time (CSISA, 2020).
	4WT bhusa Reaper	1990	The bhusa (straw) reaper is used to collect straw from fields after combine harvester use. During harvest, a combine harvester leaves residue in the field, requiring crop residue to collected – a labour-intensive and costly process. The bhusa reaper collects residues to be used for livestock feed. Farmers mostly collect wheat straw while the rice residue tends to be burned. A four-wheel tractor is required to attach the straw reaper and the reaper aggregates residue into the tractor's trolley.	Similar to combine harvesters, farmers were introduced to this technology after observing use in bordering districts of India. While there are few promotional activities for this technology, it's use may potentially reduce the level of crop residue burning in rice-wheat cropping systems of Nepal. There is anecdotal evidence of almost 100 straw reapers operational in Nepal Terai, which are primarily operated by combine harvester owners (pers comm).

### 2.2 Sampling strategy

Nepal has three distinct geographical regions: the Terai (plain area), hills, and mountains. Mechanisation options in the mountain and hills are limited due to rugged terrain. However, mechanisation is more advanced on the Nepal Terai where cropping intensity and access to markets and ground water irrigation is higher, and porous borders with India greatly influence rural economies. Therefore, the Nepal Terai was targeted for this study.

**Fig. 2:**
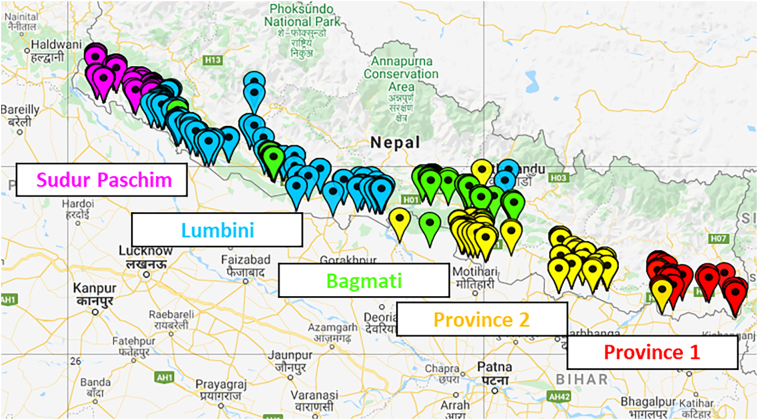
Survey respondent locations, colour coded by province.

A total of 14 districts out of 20 were purposively selected based on the higher level of interaction with development initiatives, with results likely to represent higher rates of mechanisation than other parts of the Nepal Terai. Selected districts were Nawalparasi, Rupendhai, Kapilbastu, Dang, Banke, Bardyia, Kailali, Kanchanpur, Chitwan, Bara, Parsa, Dhanusa, Sunsari, Makawanpur. The total sample size consists of 1570 farmer respondents selected randomly from each of the 14 districts (Fig. 2). Households were selected randomly by enumerators using a randomised walking strategy though each village which was purposively selected for the presence of Zero Tillage Drill service providers. The main decision-making household head was invited for interview, and participated without remuneration and if their time permitted. A structured survey questionnaire consisting of information on household demographics, cropping systems and decision making was deployed using Surveybe (https://surveybe.com/; accessed 8/25/2020). Several escape and validation rules were applied to avoid question redundancy and increase data quality.

### 2.3 Theory and calculation

Investigations of adoption are often framed within static binary ‘yes’ or ‘no’ outcomes disallowing a nuanced understanding of adoption processes, particularly at a population level whereby a singular percentage dictates progress and success (Brown et al., 2017b). To obtain a more nuanced understanding of the machinery adoption process, the PAUF (Brown et al., 2017b) was adapted. The PAUF was originally applied to understand the uptake of Conservation Agriculture practices in sub-Saharan Africa. Due to a change in focus from a multi-technology package of farmer practices (i.e. conservation agriculture) to singular practice implemented through machinery, the PAUF was modified with the removal of certain categories and replacement with relevant alternatives (Fig. 2) and named the Stepwise Process of Mechanisation (SPM) Framework. This enables a more nuanced understanding of mechanisation uptake in a population, by also including ownership components. This adaptation increases the utility of the framework and enables it to capture a slightly different adoption process for the studied context.

The SPM Framework assumes that the ultimate desirable outcome achieved by resource constrained smallholder farmers is that of unassisted use, but this is achieved though intermediate steps, and some may also attain ownership of the given machinery. Through understanding the status of machinery uptake in a stepwise process, the progression of farmers within communities (from exposure to assessment and progression and eventual utilisation decisions) can be understood and subsequent strategies formed that aim to move members of a given population from lower to higher stages of the SPM framework.

We therefore assess the status of agricultural mechanisation though classification of farmers in five phases:

1. The *Exposure* Phase that provides insights into information gaps within rural communities;
2. The *Assessment* Phase that provides insights into what happens once exposure occurs;
3. The *Continuation* Phase that provides insight into decision outcomes that occur once progression has occurred;
4. The *Utilisation* Phase that provides insights into what form of adoption is occurring; and
5. The *Ownership* Phase that provides insights into what form of ownership is occurring.

**Fig. 3:**
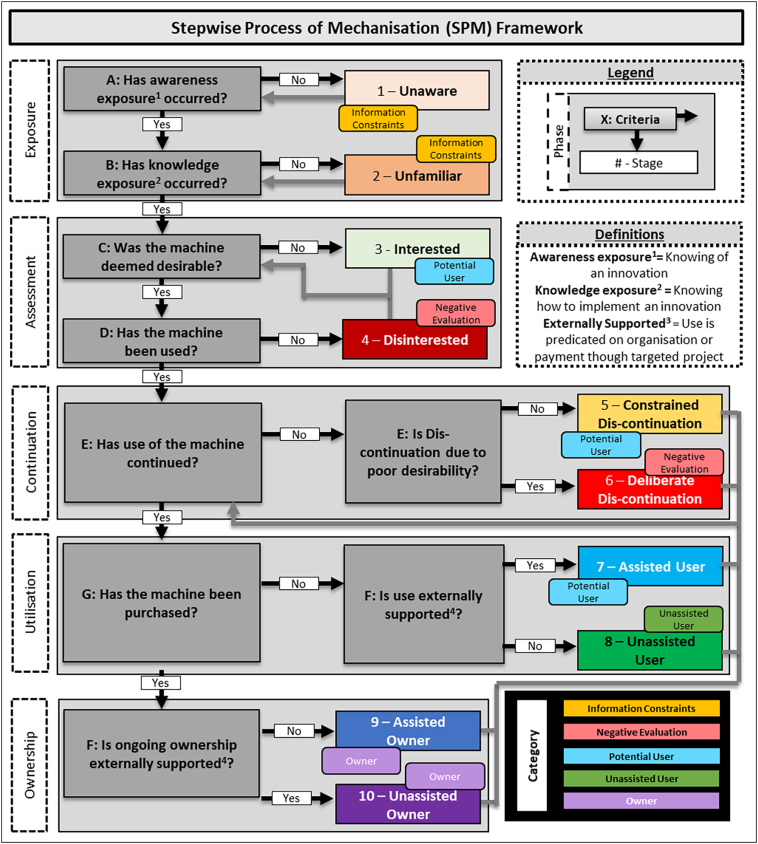
The SPM theoretical framework that enables more nuanced understanding of the status of machinery adoption in investigated populations. Note grey feedback loops facilitate the framework are dynamic, and not a static point in time. Graduation is one directional for the first two phases (i.e. once passed no regression is possible), however Question E (related to continuation) is cyclical, indicating that utilisation includes disconnection as an inevitable outcome.

**Fig. 4:**
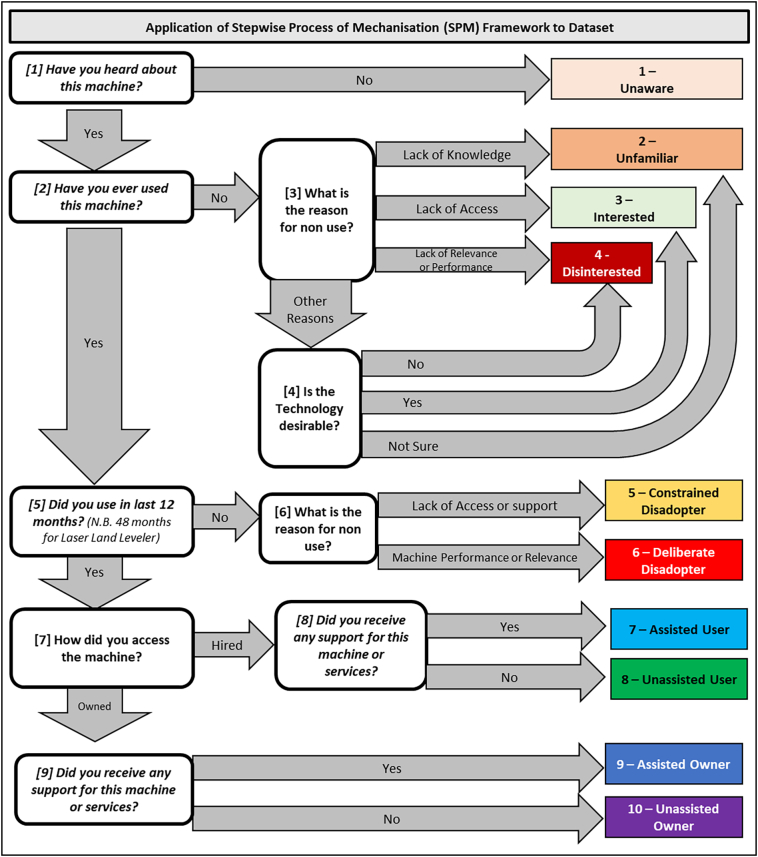
Analytical framework for implementation of the SPM Framework. Note the theoretical framework is dynamic with feedback loops (noted with grey arrows), while the analytical framework is static to capture a point in time.

A simple analytical framework was applied based on the SPM framework, to provide a tangible way to analyse collected data. An assumption is made in the quantification of exposure, with a metric of confidence of knowledge for each machine used to disaggregate unfamiliar, interested and disinterested stages. Note that ‘assisted’ refers to ongoing subsidisation by projects and is considered artificial adoption rather than as full use (Brown et al., 2017b). The only exception to the analytical framework related to the laser land leveller, where continuation (Question E; Fig. 3) was moderated with additional time, as annual use of the machine is not expected (i.e. once fields are levelled they will only need periodic maintenance across several years, and not annual use). The period for consideration of disadoption for the laser land leveller was hence changed from 12 months to 48 months. The analytical framework is provided in Fig. 4.

## 3 Results and discussion

### 3.1 Demographics of respondents

The average age of respondents was 47.8 years, of which the average length of education was 6.5 years. Each household had an average of 3.4 members participating in agriculture and 21% of households had members who had migrated elsewhere. 53% of households had credit access (formal or informal). In terms of caste, 20% were from non-marginalised caste (13% Brahmin, 7% as Chettri) and 80% were from marginalised castes (45% Aadibasi/ Janajatis, 27% Madhesi, 2% Dalits and 2% ‘other’ caste), while 4% identified as Muslim. 10% of respondents were female.

The average land holding was 1.35 ha. Only 9% identified an irrigation constraint and 88% used herbicides. In the Kharif (monsoon season), 96% of respondents planted rice (average area 1.36 ha) and 10% planted Maize. In the Spring season (Pre Kharif), 17% planted maize and 7% planted rice. In winter (Rabi), 78% planted wheat (average area 0.99 ha), 28% planted Lentil and 13% planted maize.

### 3.2 Overall status

Results indicate that the status of agricultural mechanisation in Nepal remains constrained (Table 2). As can be expected, traditional binary adoption estimates confirm that technologies with longer sensitization and promotion periods (e.g., the combine harvester and 4WT seed drill) have more advanced binary adoption rates compared to more recently introduced machinery. Yet this is not exclusively true, as the bhusa straw reaper has despite 30 years of existence in Nepal not shown substantial adoption among the surveyed population. Conversely, despite its relatively recent introduction, the 2WT power tiller reaper has achieved relatively rapid uptake.

**Table 2:**
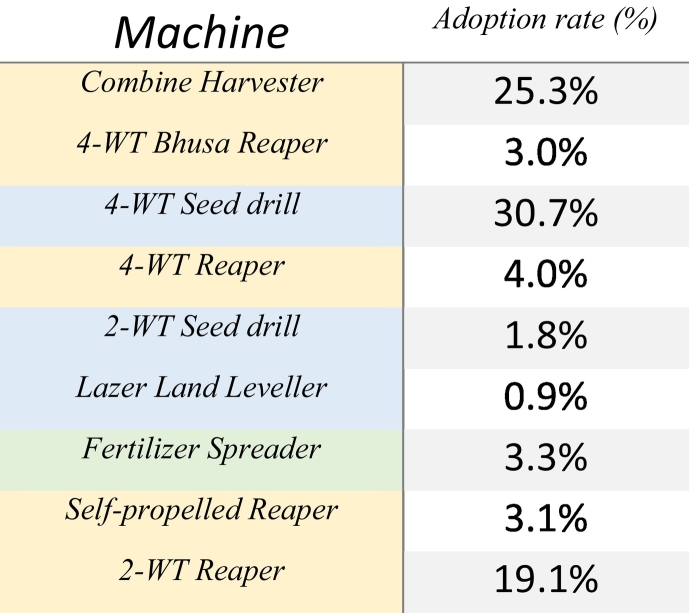
Binary rates of adoption for investigated technologies, in order from longest to most recent introduction in the Nepal Terai. Colour coding indicates categorization as planting (blue), fertilisation (green) or harvest (yellow) machinery.

**Fig. 5:**
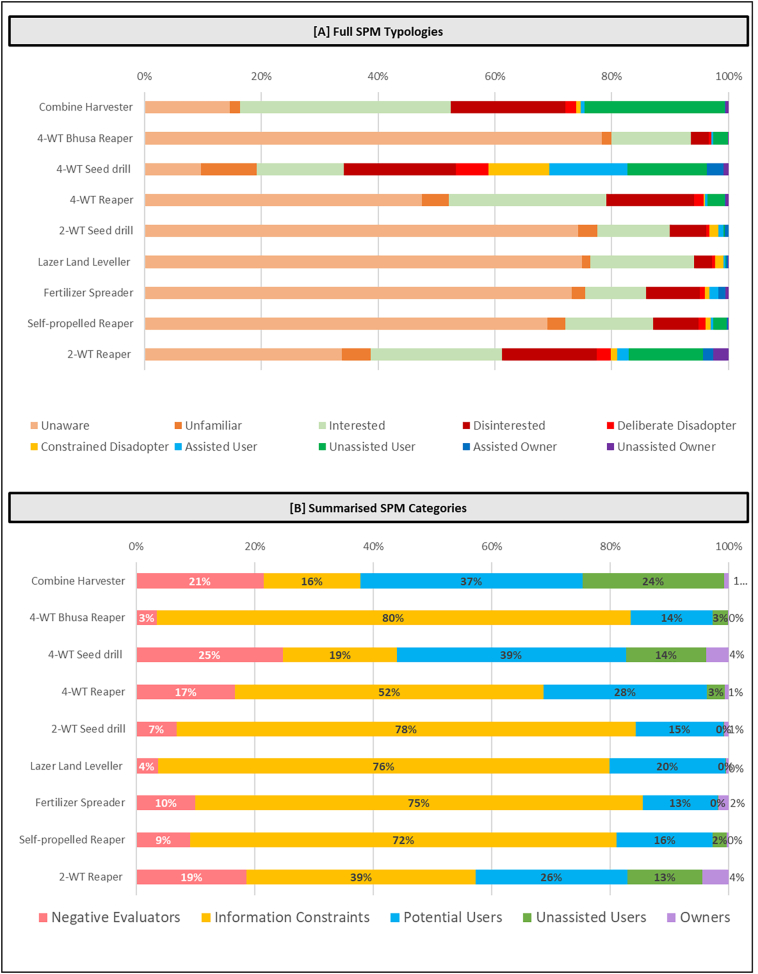
The SPM Framework output of the status of nine sustainable agricultural machines, listed in order of time since introduction in Nepal. [A] Full Typologies (top) and [B] summarised categories (bottom) are provided. *n* = 1569 for all machines.

Such binary statistics are where many adoption studies tend to stagnate (Brown et al., 2017b). However, the SPM Framework provides a more nuanced contextualisation of the binary statistics presented (Fig. 5). From this, it can be deduced that for most machines there exists a substantial information gap, while progression to sustained use once information is obtained is constrained. The two exceptions appear to be the four-wheel tractor 4WT driven seed drill and the combine harvester (both with three decades of use in Nepal) that show comparatively high exposure and progression rates. Promoting bodies (e.g. through government and development programs) will often look for ‘low hanging fruit’ in the form of potential users who express interest or are in an experimentation with a given technology, yet for many investigated machines, the pool of potential users is comparatively small (e.g., 4WT Bhusa Reaper), which highlights the need for alternative strategies compared to where the pool of potential uses is larger (e.g., 2WT reaper). Likewise, substantial negative evaluation paired with comparatively high current use (e.g., 4WT seed drill) will dictate the need for an alternative strategy compared to where current use is limited, and information gaps dominate in the population (e.g., laser land leveller and Bhusa Reaper). As fee-for use service provision is integral in smallholder systems, attention is also provided to ownership, though as expected this is mostly limited. These learnings are further explored through the five phases of the SPM framework in the following sections.

### 3.3 Stagnation at the exposure phase

The exposure phase identifies the proportion of the population with insufficient information to evaluate each machine (i.e.*, ‘Unaware’ and ‘Unfamiliar’ stages*) compared to those who have sufficient information to evaluate each machine (i.e.*, the remaining stages*) to understand if awareness is a core constraint to the adoption process. Lower exposure suggests that movement along the adoption pathway may potentially be constrained by information flows, leading to a focus on informational promotion strategies that enable communities to access information, learn about potential innovations and assess the potential benefits of different mechanisation options. It is the first step passed in initially learning of any innovation.

Overall, there were substantial information gaps identified, with five of the nine technologies found to have a non-exposure rate in the surveyed population above 70% (yellow segments in Fig. 5B). In only two cases (Combine harvester and 4 WT Seed Drill) was the level of non-exposure below one in five. While five of the investigated technologies had less than a decade of existence in Nepal, results indicate that overall low rates of exposure are not solely related to time. This is exemplified by the experience of the 2WT reaper, with only six years of presence in Nepal but achieving 61% exposure within the surveyed population.

In seven cases unawareness was the dominant component of the non-exposure rate, indicating very limited recognition of investigated machinery. This is consistent when considering each household's overall exposure is considered across the nine machines. 66% of households were either unaware or unfamiliar with at least five of the machines investigated, while one quarter of respondents were unaware of unfamiliar with at least seven of the machines investigated. Only 3% of respondents were familiar with all nine machines, and only 13% of households were familiar with at least three of the investigated machines. This highlights that there are substantial information gaps that are constraining agricultural mechanisation broadly in the investigated communities.

**Fig. 6:**
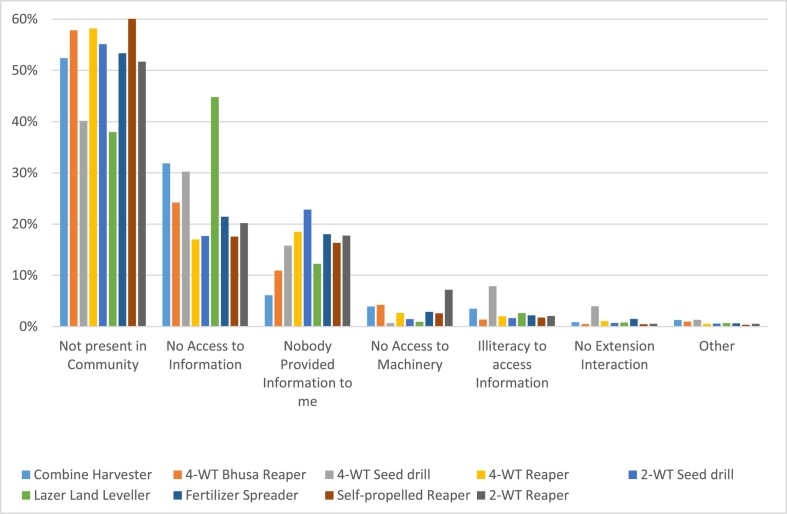
Reasons for stagnation at the exposure phase for the nine investigated technologies.

The primary self-identified reason for a lack of exposure to each technology was that that ‘no farmers in the area use that machine’ (53% of all responses by those with limited exposure, and the primary reason for all machines except the laser land leveller where the primary reason was ‘no information access’; Fig. 6). This highlights the importance of the ‘see to believe’ mentality of farmers in learning of new machinery and innovations, enforced with 58% of those with information gaps identifying ‘neighbours and nearby farmers’ as their main source of information. Groups and cooperatives (13%) and mass media/ Jingling (8%) were the other dominant forms of information, while only 6% identified Projects an NGOs as their main source of info, and only 2% identified government extension. This may suggest that public extension may not be the best information pathway to intensify over the short term, given limited current recognition by respondants.

In cases where adoption is present yet there is an identification of a lack of other farmers undertaking a technology (e.g. Combine harvester), this may be a reflection of the complex social hierarchy present in South Asia whereby caste dictates access to information and interaction outside of limited social circles (Aryal and Holden, 2013; Krishna et al., 2019). Additionally, the limited identification of Government, NGO and project influence in obtaining information may also reflect social hierarchy dictating access to information, alongside the current extension void present due to the rapid transformation from a central to local government extension mandate in line with the 2015 constitution. In this process, district-level agricultural officers have been disbanded and new systems to enable agricultural extension are still being developed, though those that have been formed have been found to have limited capacity (Thapa et al., 2019; Yilmaz et al., 2020). Babu and Sah (2019) have previously shown that the capacity of human resources, infrastructure, financial funds and other resources is currently insufficient to enable a successful extension system. Studies on extension officers' degree of knowledge and resulting extension capacity in Nepal on appropriate machinery are lacking, highlighting an important research and development need. Outside of formal extension systems, other considerations may include the permeation of technologies from India. Indian agriculture also has a substantial influence on the Nepal Terai and it is likely that technologies not prevalent on the Indian Gangetic Plains may lead to slower exposure rates in Nepal. For example, the fertilizer spreader is more prominent in China and is not widely present in India, and likewise 2WT operated reapers and seed drills are more prevalent in Bangladesh than India (Van Loon et al., 2018). This also relates to machinery availability, where machines such as the combine harvester cross the border, reducing the need for Nepali investment in machinery to access services. This is less likely with smaller, 2WT machinery which has to date had more limited Nepali ownership and investment.

### 3.4 Limitations in moving past the assessment phase

The assessment phase provides insights into what happens once exposure has occurred, and a household has enough information to make an assessment. This is represented through the removal from analysis of respondents who remain in the exposure phase, leaving three outcomes possible at this phase: interest, disinterest, and progression to use. Promoters of an innovation typically aim to reduce the time individuals spend in this phase and aim for progression to use in a speedy manner. Substantial dis-interest (i.e., pre-use negative evaluation) suggests a lack of contextual relevance or obvious benefit to (certain) users, meaning it may be necessary for the machine to undergo further modification to increase relevance or benefit. Substantial interest (i.e., pre-use positive evaluation without progression) suggests either a relatively new machine or constraints in implementation despite perceived benefits (e.g., financial or machinery availability constraints), which may suggest a need for subsidisation or other incentives to overcome costs or increases in availability of machines. In such cases, qualitative research is just as important as quantitative adoption studies and should be implemented to understand decision progresses, a void which is still evident in the literature. A substantial proportion of the population progressing to use suggests that machines are positively evaluated and accessed with limited constraints.

**Fig. 7:**
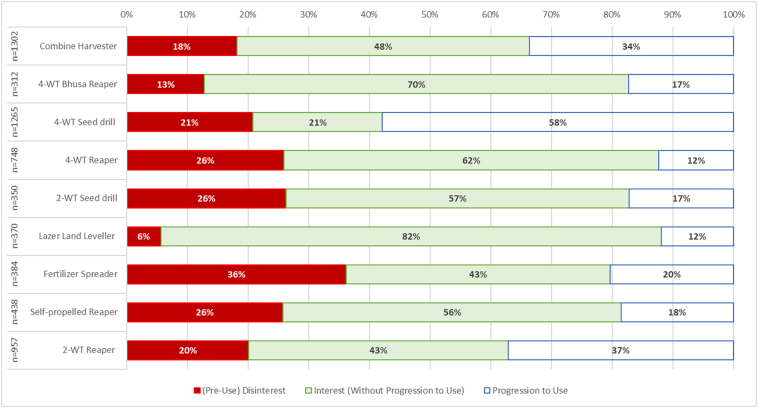
Progression Phase for nine investigated machines, which highlights the way in which members of the population have applied knowledge on each machine. The left-hand column provides the number of respondents presented for each machine, which excludes from analysis those who remain in the exposure phase.

Results suggest that the proportion of disinterest across investigated machinery was limited, indicating that predominantly, machines have perceived potential benefits to respondents (Fig. 7). In eight of the nine machinery investigated, interest without progression (i.e. positive perception of machinery without progression) was dominant (expectation being 4WT Seed Drill where Progression to use was dominant). The dominant reason for interest without progression was a lack of service providers, which accounted for above 80% for all machines. This suggests the machinery access is the major inhibitor to further mechanisation on the Nepal Terai. Despite limited disinterest, there was a substantial lack of progression in all studied machineries except the 4WT seed drill (with a 3 in 5 progression ratio, likely reflecting a longer dissemination time in existence in Nepal). Six of the nine technologies had a progression ratio below 1 in 5.

**Fig. 8:**
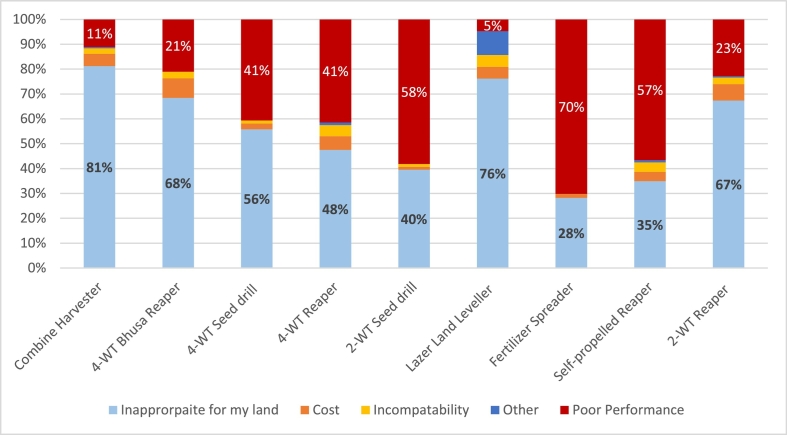
Reasons identified for disinterest in each of the nine technologies.

Where pre-use disinterest did occur, the primary reason in all but to machines (Spreader and 2WT Seed Drill) was inappropriate land size, primarily the perception that land was too small to accommodate the machine in question (Fig. 8). As this study investigated both two- and four- wheel equipment, as well as hand powered equipment and the large combine harvester, this is to be expected. This does also tend to confirm with a growing literature body on the need for land consolidation to achieve intensification in South Asia (Niroula and Thapa, 2005; e.g., Dhakal and Khanal, 2018). Poor performance was substantial (>40% of disinterested respondents) in five of the machines investigated, suggesting that exploring further the reasons for poor performances are warranted. Cost was however not a driving factor of disinterest.

Not all technologies will suit all members of the community and some dis-interest is an expected outcome. With limited disinterest identified across the investigated machinery, future progression from positive evaluation to use may be likely to occur. Comparatively, the only other study to use a similar framework (Brown et al., 2017b) found substantial rates of disinterest in minimum tillage (between 20% and 65% depending on country) and in the majority of cases at least 25% disinterest across three Conservation Agriculture based practices in five sub-Saharan African countries. Contrary to the conclusion that future uptake of CA in Africa may remain limited, these results suggest that the pool of potential adopters is considerable, and uptake is likely to increase over the short to medium term, if the identified constraints are addressed.

### 3.5 Disadoption at the continuation phase

The continuation phase is crucial to understanding the success of a used innovation because it reflects the rate in which a machine is successful for users and the community at a particular point in time. To do this, analysis in this phase removes those who have not graduated to use (i.e. the exposure and assessment phases). A higher disadoption rate suggests issues in implementation, or in the case of aging innovations it may suggest obsolescence as new innovations supersede existing practices. Disadoption may be deliberate or due to constraining factors and this is important in understanding the future re-adoption potential of investigated machines.

**Fig. 9:**
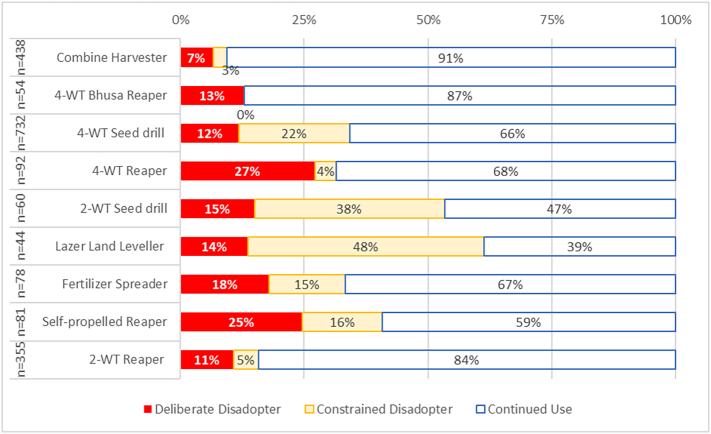
The Continuation Phase for nine investigated machines, which excludes respondents who have not used the machinery (i.e. exposure and assessment phases) to visualise what happens once use of a machine occurs. The left-hand column provides the number of respondents presented for each machine.

Results suggest issues in implementation with the majority of investigated machines. Six of the nine machines had a dis-adoption rate near or above one in three, with only the combine harvester, Bhusa reaper and 2WT reaper showing comparatively low disadoption rates. The 2WT seed drill and laser land leveller had greater than 1 in 2 disadoption rate (Fig. 9). Disadoption rates tended to be higher for planting machinery, as opposed to harvest machinery.

Despite substantial disadoption rates, planting machinery (both 2WT and 4 WT seed drills and the laser land leveller) was dominated by constrained disadoption. This likely relates to the high level of subsidized adoption (see Section 3.6) that can lead to temporary adoption that was not sustained once projects and the artificial enabling environments and promotional efforts cease, particularly with exiting project aligned service providers. For the remaining machinery, deliberate disadoption was dominant, driven primarily by experiences of poor performance. Land suitability, primarily for low-lying and waterlogged rice plots during the harvesting time was cited as a reason for combine harvester dis-adoption (48%), and likewise land suitability was the reason for 36% of disadoption for the hand spreader, likely due to larger plot sizes.

### 3.6 Subsidisation rates in the utilisation and ownership phases

The utilisation phase is crucial to understanding how an innovation is currently being used, and excludes those not currently using the machinery in requestion (i.e. exclusion of all prior phases from this phases' analysis). For instance, a high proportion of assisted users may indicate project driven adoption and the potential for steeper disadoption rates once incentives end (Brown et al., 2017a; As discussed in Section 3.5). Likewise, ownership indicates a deeper commitment to the machine and indication of likely sustained use, as well as the potential to provide machinery-for-hire services to other farmers in their communities.

**Fig. 10:**
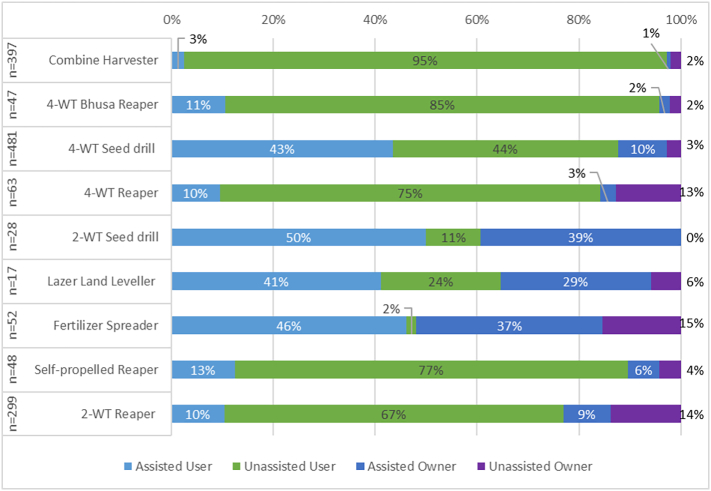
Utilisation Phase for nine investigated machines, which excludes all prior phases to focus on current elements of utilisation of the investigated machines. The left-hand column provides the number of respondents presented for each machine, noting that use and hence sample size is constrained for some machinery.

Fig. 10 highlights that for planting and fertilising machinery, the majority of use and ownership for each of the four machines is through subsidized programs. This may indicate that true or sustained adoption is not assured once artificial incentives are removed (Brown et al., 2020). This has been a commonly observed practice with minimum tillage planting practices globally (Giller et al., 2009; Andersson and D'Souza, 2014; De Roo et al., 2019) where ‘pseudo-adoption’ is commonly attributed as adoption yet ceases when incentives finish and is seen in the continuation phase of this investigation (Kiptot et al., 2007).

This was not the case with harvest equipment – and particularly for combine harvesters – which tended to have a comparatively higher proportion of unassisted users, and never more than 13% assisted use. This reflects significantly less engagement of projects and NGOs with these machines; in the case of combine services, many of the machines used in the Nepal Terai are migrated seasonally from India to harvest rice and wheat (CSISA, 2020). Despite this, ownership remained constrained across most technologies, with the exception of the fertilizer spreader (a low-cost machine that does not have a service provision element) and the 2WT reaper (likely as this attachment is a cost-effective addition to an already purchased 2WT). However, ongoing support for ownership was dominant for planting and fertilising equipment in particular, highlighting potential longer-term constraints once support ceases.

Given that rates of ownership were below 2% for seven of the nine machines evaluated, that 80% interested respondents indicated machinery access was the reason for a lack of progression and constrained adoption was substantial in five machines and dominant in all three planting machinery, it can be argued that the focus Nepal has placed on subsidies does not appear to have led to substantial unassisted ownership of machinery, and certainly not at levels where easy access to machinery abounds within communities. Such results may reflect that large and widespread subsidies may not always be the best way to promote broad agricultural mechanisation. Even with substantial subsidies, large and expensive equipment such as combine harvesters and laser land levellers (which require higher horsepower 4WT) are unlikely to reach substantial levels of private ownership and may be best advanced through co-operatives and custom hire centres where costs are shared more widely, or by governments. For 4WT and 2WT attachments, private ownership may be more achievable, due to lower costs compared to prior investments made on tractors (the more expensive component compared to attachments). This could form a greater focus for promotional programs, alongside targeted training programs for existing and potential machinery owners. In terms of individual machinery ownership and service provision, questions still remains on what can be done to ensure economic viability of agricultural service provision, particularly when agricultural service provision rarely can compete with other non-agricultural tractor service provision activities (Pradhan et al., 2016). Overall, a knowledge gap exists on how to foster increased small scale and localised service provision in the literature with a particular focus on the lived experiences of service providers. This warrants further investigation and deeper understanding.

## 4 Conclusions

For the first time in South Asia, a systematic analysis through a novel stepwise framework has clarified and updated the status of agricultural mechanisation on the Nepal Terai, and particularly the status of mechanisation beyond a metric involving tractor abundance. This has highlighted that current attempts spanning decades to scale agricultural machinery have not (yet) achieved their intended outcomes. There does however appear to be hope for the near future, with the existence of substantial numbers of potential users who could be quickly transitioned to use, particularly if supply-side machinery constraints can be overcome. This is evidenced by the rather rapid uptake of the 2-wheel tractor reaper in a short period of time, and likewise the uptake of harvest machinery with minimal government intervention. Closing large exposure gaps is also likely to increase that pool of potential adopters if the same rate of interest continues for those gaining exposure to the investigated machinery. If further institutional and governance barriers can be overcome (especially the revision of demonstration, extension and subsidy programs and their influence on ownership outcomes), mechanisation in Nepal may be able to mimic other quickly mechanising rural economies like Myanmar (Belton et al., 2021).

Given the increased nuance in understanding of the process and status of mechanisation gained through this approach, the SPM framework provides a first step to understanding a broader scaling process. Both in Nepal and beyond, future work could be applied to understand elements of responsible scaling (especially in understanding if particular strata of communities tend to be over-represented as certain SPM typologies or tend not to progress into certain stages, such as based on religion, gender, financial endowments and/or caste). This addresses a need established by Wigboldus et al. (2016) to apply analytical tools that ensure responsible scaling of technologies and practices. Combining this framework as a basis for future scaling investigations, particularly ex-post investigations, will also help build nuanced understanding around farmer decision making and inertia that forms elements of many proposed scaling frameworks (e.g. Wigboldus et al., 2016; Sartas et al., 2020; Kernecker et al., 2021). This could be particularly powerful when paired with in depth qualitative explorations of farmer decision making (e.g. Brown et al., 2017a; Brown et al., 2021). Such work is relevant not just to Nepal but to the emerging debates around the possibilities for agri-mechanisation across other emerging rural economics, particularly in South Asia and sub-Saharan Africa. Used in conjunction with socio-political, cultural and environmental explorations, development of promotional and extension strategies can become more nuanced to ensure sustainable, equitable and fast-paced agri-mechanisation based development.

## Funding

This Study was co-supported by four research initiatives: The Sustainable and Resilient Farming Systems Intensification in the Eastern Gangetic Plains (SRFSI) project (Australian Centre for International Agricultural Research - ACIAR project CSE/2011/077); the 10.13039/100000200Cereal System Initiative for South Asia (CSISA) Project (United States Agency for International Development (USAID) [Grant no: BFS-G-11-00002]) and 10.13039/100000865Bill & Melinda Gates Foundation (BMGF) [Grant no: OPP1133205]); and the ‘Roadmaps for Sustainable Agricultural Mechanisation in Nepal’ (Roadmaps) project (ACIAR project WAC/2018/220). SRFSI, Roadmaps and CSISA are implemented by CIMMYT of the CGIAR.

## Ethics approval

This research was approved by the CIMMYT ethics committee (IREC 2019.021).

## Declaration of Competing Interest

The authors have no relevant financial or non-financial interests to disclose.
